# Comparison of Sacubitril/Valsartan Versus Enalapril in the Management of Heart Failure

**DOI:** 10.7759/cureus.16332

**Published:** 2021-07-12

**Authors:** Shehar Bano, Pooja Bai, Sameet Kumar, Nomesh Kumar, Ahmed Ali, FNU Pariya, FNU Versha, Dua Khalid, Haya Khalid, Amber Rizwan

**Affiliations:** 1 Internal Medicine, University of Health Sciences, Lahore, PAK; 2 Internal Medicine, Liaquat University of Medical and Health Sciences, Jamshoro, PAK; 3 Internal Medicine, Chandka Medical College Larkana, Karachi, PAK; 4 Internal medicine, Liaquat University of Medical and Health Sciences, Jamshoro, PAK; 5 Infectious Diseases, University of Louisville, Louisville, USA; 6 Internal Medicine, Jinnah Sindh Medical University, Karachi, PAK; 7 Family Medicine, Jinnah Postgraduate Medical Center, Karachi, PAK

**Keywords:** hear failure, hospitalization, outcome, enalapril, saccubtril/vaslartan

## Abstract

Background and objective

The recent emergence of new molecules like angiotensin receptor-neprilysin inhibitor (ARNI) has highlighted the need for an update in heart failure (HF) management, as they have proven to yield better patient outcomes compared to the traditional angiotensin-converting enzyme inhibitor/angiotensin II receptor blocker (ACEI/ARB) use. This study aimed to compare HF-related hospitalization and death in patients on either ACEI/ARBs or ARNI in a local setting.

Methods

This two-arm interventional study was conducted in the cardiology and internal medicine units of a tertiary care hospital in Pakistan from July 2018 to December 2020. After enrollment, participants were randomized into two groups as per 1:1 ratio using an online research randomizer software (https://www.randomizer.org). Group A received 24/26 or 49/51 mg sacubitril/valsartan twice daily for HF. Group B received 2.5 or 5 mg enalapril twice daily. Patients were followed up for 12 months or till the development of an event.

Results

The sacubitril/valsartan group had significantly fewer HF-related hospitalizations compared to the enalapril group (13.8% vs. 22.4%; p-value: 0.03), with a relative risk reduction (RRR) of 38.3%. The sacubitril/valsartan group had 52% RRR for HF-related deaths compared to the enalapril group.

Conclusion

Based on our findings, treatment with sacubitril/valsartan was superior to enalapril in reducing the risk of hospitalization and death related to HF. The magnitude of the beneficial effects of sacubitril/valsartan as compared to enalapril on cardiovascular mortality was at least as high as that of long-term treatment with enalapril.

## Introduction

Heart failure (HF) is a clinical syndrome, which is characterized by the failure to maintain cardiac output necessary to meet the oxygen demands of the body due to dysfunctional cardiac muscles and neurohormonal activity [1,2]. It is a progressive condition with no cure and requires life-long management with a combination of drugs and devices to reduce morbidity. It affects approximately 40 million people worldwide and has a one-year mortality and hospitalization rate of 7.2% and 31.9%, respectively [1,3]. The prevalence of HF increases after 60 years of age, particularly in those with coronary artery disease, hypertension, and other cardiac comorbidities [4,5].

The most common symptoms in HF are dyspnea, orthopnea, fatigue, reduced exercise tolerance, ankle swelling, and other symptoms of fluid overload [4]. Based on the severity of these symptoms, the disease is classified into four categories according to the New York Heart Association (NYHA) functional classification, ranging from class I with no limitation to normal physical activity to class IV with severe limitations and symptoms at rest [4,5].

The mainstay of medical management for HF entails the inhibition of the renin-angiotensin-aldosterone system (RAAS), sympathetic nervous system, and neprilysin [6]. The detrimental upregulation of the RAAS mediates the pathophysiology and progression in HF, leading to fluid retention, vasoconstriction of the peripheral arteries, hypertrophy, and remodeling of the cardiac tissue [7]. For this reason, angiotensin-converting-enzyme inhibitors (ACEIs) and angiotensin II receptor blockers (ARBs) have been recommended as the mainstays of management of the condition. Several studies have found that the inhibition of the RAAS by either ACEIs or ARBs considerably reduces morbidity and mortality in HF [8-11].

The recent indication of the role of angiotensin receptor-neprilysin inhibitor (ARNI) has highlighted the need for an update in HF management. Neprilysin is a proteolytic enzyme that breaks down several peptides essential for the regulation of renal and cardiovascular metabolism and homeostasis. Inhibition of this pathway by ARNI has proven to yield better patient outcomes as compared to the traditional ACEI/ARB use [6,12]. The Prospective Comparison of ARNI with ACEI to Determine Impact on Global Mortality and Morbidity in Heart Failure (PARADIGM-HF) trial, which compared the effects of enalapril (ACEI) and sacubitril/valsartan (ARNI) on chronic HF patients, found that compared to enalapril, sacubitril/valsartan showed a better reduction in cardiovascular mortality and HF-related hospitalizations in patients with chronic HF [6,13].

There is no local data available comparing the efficacy of conventional methods with that of new treatment options for HF. In light of that, the objective of this study was to compare HF-related hospitalization and death in patients on either ACEI/ARBs or ARNI in a local setting.

## Materials and methods

This longitudinal study was conducted in the cardiology and internal medicine units of a tertiary care hospital in Pakistan from July 2018 to December 2020. The ethical review board approval was obtained before the enrollment of patients. After obtaining informed consent, 400 treatment-naïve patients with HF were enrolled in the study via consecutive convenient non-probability sampling. Diagnosis of HF was made based on the clinical presentation of shortness of breath, weakness, and edema, reduced left ventricular ejection fraction (<40%), and elevated N-terminal pro-B-type natriuretic peptide (NT-proBNP). The cut-off value for NT-proBNP for patients aged <50 years was 450 pg/mL, and it was 900 pg/mL for patients ≥50 years in age [14].

After enrollment, the participants were randomized into two groups as per 1:1 ratio using an online research randomizer software (https://www.randomizer.org). Group A received 24/26 or 49/51 mg sacubitril/valsartan twice daily for HF. Group B received 2.5 or 5 mg enalapril twice daily. The decision on dosing was made based on the severity of the symptoms.

The patients' characteristics such as age, gender, history of smoking, blood pressure, previous history of myocardial infarction, ejection fraction, and NT-proBNP were recorded in a self-structured questionnaire. Patients were followed up for 12 months or till the development of an event. The event was defined as hospitalization or death related to HF.

The number of participants lost to follow-up in the sacubitril/valsartan and enalapril group was 19 and 17, respectively. The final analysis included only those participants who completed the study. Statistical analysis was performed using the SPSS Statistics version 22.0 (IBM, Armonk, NY). Continuous variables were analyzed via descriptive statistics and were presented as mean and standard deviation (SD). Categorical variables were presented as percentages and frequencies. Relative risk (RR) and relative risk reduction (RRR) were calculated via an online calculator (MedCalc) using a 95% confidence interval. A p-value of less than 0.05 meant that there is a significant difference between the two groups and the null hypothesis is void.

## Results

The characteristics between both groups were comparable, indicating that our randomization was successful (Table 1).

**Table 1:** Characteristics of the participants SD: standard deviation; BMI: body mass index; NT-proBNP: N-terminal pro-B-type natriuretic peptide

Characteristics	Sacubitril/valsartan group (n=181)	Enalapril group (n=183)	P-value
Age in years (mean ± SD)	53 ± 12	55 ± 12	0.12
Male, n (%)	88 (48.62%)	90 (49.18%)	0.91
Hypertension, n (%)	165 (91.16%)	170 (92.90%)	0.54
Smoking, n (%)	52 (28.73%)	55 (30.05%)	0.78
Diabetes, n (%)	68 (37.57%)	65 (35.53%)	0.68
Hypercholesterolemia, n (%)	100 (55.25%)	106 (57.92%)	0.60
BMI greater than 25 kg/m^2^,n (%)	56 (30.94%)	50 (27.32%)	0.44
Ejection fraction, %, (mean ± SD)	31.21 ± 4.12	31.82 ± 4.02	0.10
NT-proBNP (pg/mL), (mean ± SD)			
Age below 50 years	542.12 ± 50.12	551.22 ± 48.52	0.07
Age 50 years and above	1201.23 ± 101.34	1211.61 ± 99.98	0.32

The sacubitril/valsartan group had significantly fewer HF-related hospitalization compared to the enalapril group (13.8% vs. 22.4%; p-value: 0.03). The RRR was 38.3%. The sacubitril/valsartan group had a 52% RRR for HF-related death compared to the enalapril group; however, the difference was not significant (Table 2).

**Table 2:** Comparison of HF-related events between sacubitril/valsartan and enalapril groups HF: heart failure; RR: relative risk; RRR: relative risk reduction; CI: confidence interval

Events	Sacubitril/valsartan group, n (%), (n=181)	Enalapril group, n (%), (n=183)	RR (95% CI)	RRR (%)	Number needed to treat	P-value
HF-related hospitalization	25 (13.8%)	41 (22.4%)	0.61 (0.39-0.97)	38.3%	11.63	0.03
HF-related death	7 (3.8%)	15 (8.1%)	0.47 (0.19-1.12)	52%	23.1	0.09

## Discussion

The findings of our study demonstrated that sacubitril/valsartan yielded superior results as compared to enalapril in treating HF. Results of our study are consistent with other studies as similar superiority has been shown by several other studies. McMurray et al. have concluded that the inhibition of angiotensin II receptors with sacubitril/valsartan was comparatively more effective than that with enalapril in terms of RR of cardiovascular causes and hospitalization for HF [13].

Neprilysin, a neutral endopeptidase, degrades several endogenous vasoactive peptides, including the natriuretic peptides, bradykinin, and adrenomedullin [15]. Increased levels of these substances secondary to neprilysin inhibition cause neurohormonal over-activation, resulting in vasoconstriction, sodium retention, and maladaptive remodeling [16,17]. Combined inhibition of the RAAS and neprilysin has shown better results, but the clinical trials have shown serious angioedema after combined inhibition of ACEI and neprilysin [18]. Sacubitril/valsartan, consisting of neprilysin and ARB inhibitor, decreases the risk of angioedema. It is superior to enalapril in reducing the risk of mortality from any cause and also in terms of physical limitations of HF [13]. Treatment with sacubitril/valsartan led to symptomatic hypotension secondary to its vasodilator effects; however, no increase in the rate of discontinuation was observed due to this adverse effect. Impaired renal perfusion, increased serum creatinine level, and drug discontinuation due to renal impairment were more commonly seen in the enalapril group than the sacubitril/valsartan group [13]. The mainstay of HF therapy includes prevention of the worsening of HF and maintaining clinical stability. The mortality benefits with sacubitril/valsartan were greater as compared to enalapril. Compared with enalapril, the risk of hospitalization for HF and overall hospitalization for any cause with sacubitril/valsartan was reduced by 23% and 16%, respectively [19]. Valsartan has 60% greater bioavailability when administered as sacubitril/valsartan than as a single-agent formulation. Thus, the 103 mg of valsartan present in a 200 mg dose of sacubitril/valsartan provides an equivalent systemic exposure to the 160 mg dose of valsartan, which is the maximum twice-daily recommended dose for the treatment of HF [19].

To the best of our knowledge, this is the first study in a local setting on the role of sacubitril/valsartan in reducing HF-related complications and death. However, since the study was conducted at a single institute, the sample was less diverse and limited. The impact of other parameters like echo findings and electrocardiogram changes could not be assessed due to limited resources.

## Conclusions

As per our findings, treatment with sacubitril/valsartan was superior to enalapril in reducing the risk of mortality and hospitalization secondary to HF. The magnitude of the beneficial effects of sacubitril/valsartan as compared to enalapril on cardiovascular mortality was at least as high as that of long-term treatment with enalapril. However, further large-scale multi-centric studies are needed to confirm the results of our study.
